# Reducing Crowding by Weakening Inhibitory Lateral Interactions in the Periphery with Perceptual Learning

**DOI:** 10.1371/journal.pone.0025568

**Published:** 2011-10-31

**Authors:** Marcello Maniglia, Andrea Pavan, Luigi F. Cuturi, Gianluca Campana, Giovanni Sato, Clara Casco

**Affiliations:** 1 Department of General Psychology, University of Padua, Padua, Italy; 2 Cognitive Neuroscience Sector, SISSA, Trieste, Italy; 3 Integrated Centre for Research and Treatment of Vertigo, Balance and Ocular Motor Disorders, University Hospital Munich-Großhadern, Munich, Germany; 4 Centro di Riabilitazione Visiva Ipovedenti c/o Istituto L. Configliachi, Padua, Italy; McMaster University, Canada

## Abstract

We investigated whether lateral masking in the near-periphery, due to inhibitory lateral interactions at an early level of central visual processing, could be weakened by perceptual learning and whether learning transferred to an untrained, higher-level lateral masking known as crowding. The trained task was contrast detection of a Gabor target presented in the near periphery (4°) in the presence of co-oriented and co-aligned high contrast Gabor flankers, which featured different target-to-flankers separations along the vertical axis that varied from 2λ to 8λ. We found both suppressive and facilitatory lateral interactions at target-to-flankers distances (2λ - 4λ and 8λ, respectively) that were larger than those found in the fovea. Training reduces suppression but does not increase facilitation. Most importantly, we found that learning reduces crowding and improves contrast sensitivity, but has no effect on visual acuity (VA). These results suggest a different pattern of connectivity in the periphery with respect to the fovea as well as a different modulation of this connectivity via perceptual learning that not only reduces low-level lateral masking but also reduces crowding. These results have important implications for the rehabilitation of low-vision patients who must use peripheral vision to perform tasks, such as reading and refined figure-ground segmentation, which normal sighted subjects perform in the fovea.

## Introduction

A widely used model of early visual processing suggests that the retinal image is encoded by mechanisms that respond locally and independently to a specific range of orientations and spatial frequencies [1]–[3].

In the last two decades, a large body of psychophysical and physiological evidence has suggested that these mechanisms do interact, although they are assumed to be local and independent. A number of studies have corroborated this evidence by showing that the contrast threshold for detecting a target (either a Gabor patch or a bar) was modulated if the target was flanked by two high-contrast Gabor patches or bars [4]–[6]. Whether the flankers reduced or increased contrast thresholds depended on their relative orientation and distance with respect to the target. It has been shown that the contrast threshold of Gabors presented in fovea decreases in the presence of co-oriented and co-aligned (collinear) flankers [4]–[8]. This facilitation is maximal for a target-to-flankers separation of approximately three times the Gabor carrier wavelength (3λ). On the other hand, smaller separations can increase the target contrast threshold, depending on the flankers' contrast and the phase of the cosinusoidal carrier [9]. Complementary physiological data have suggested that the substrate of these spatial interactions may be found at the early level of visual processing [8], [10]–[17].

This pattern of lateral interactions between early cortical neurons, which results from different target-to-flankers distances, can be modulated by practicing target contrast detection through a process termed perceptual learning [18]. In particular, the strong lateral suppression observed in an abnormal pattern of connectivity (such as in amblyopia) has been shown to disappear and to be replaced by some facilitation after training [18]. Perceptual learning has been shown to be specific for the low-level trained stimulus and for the task, which suggests modifications of neural processes at the level of the striate cortex in adults. However, systematic training in this low-level task also seems to yield significant perceptual benefits to unrelated visual functions, such as visual acuity, that may share the same linear filtering at an early stage of processing [18]–[20].

To date, most investigations of the pattern of lateral interactions as well as their modulation by perceptual learning and the transfer of low-level learning to high-level tasks have been conducted with stimuli presented in the fovea. When the stimulus position is off-fixation (e.g., from 1° to 4° eccentricity), there is failure in finding consistent collinear facilitation [21]–[24] despite the fact that the stimuli are M-scaled. At 4° eccentricity and spatial frequencies of 3–4 cpd, one study found inhibition with collinear flankers [23] whereas another study found facilitation [24] that was larger with orthogonal flankers than with collinear flankers. Furthermore, it is unclear whether perceptual learning modulates lateral interactions in the periphery. Fittingly, previous results are not consistent [24], probably because the number of sessions used was insufficient [25].

In the present study, we investigated lateral interactions in the periphery and whether these can can be modulated by training the contrast detection of a flanked target, either by reducing the inhibitory or by strengthening the facilitatory lateral interactions between the target and flankers. We also asked whether the training effect transfers to different orientations and different retinal positions. Moreover, we explored whether training-dependent reduction of low-level inhibitory lateral masking could reduce a peripheral masking effect known as crowding [26]–[28], whereby a stimulus is presented with flankers that generally decrease the visual acuity for that stimulus [26], [29]. In fact, although inhibition of contrast detection and crowding are two distinct phenomena [26], [30], they may share the same first stage of linear filtering [29].

**Figure 1:**
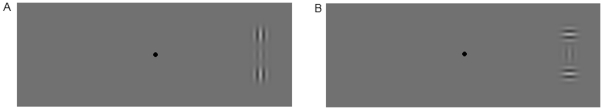
Stimuli used in the experiments. (A) Collinear configuration: the target (central patch) has the same orientation as that of the flankers. (B) Orthogonal configuration: the flankers are oriented horizontally with respect to the central vertical target. In this example, the stimuli are located to the right with respect to the fixation point (4° eccentricity), and their position was randomized across trials. The stimuli here have a spatial frequency of 4 cpd, and the target-to-flankers distance is 3λ. The target (central patch) has a lower contrast than the flankers. For illustrative purposes, the Gabor patches here have exaggerated contrast.

In order to determine baseline performances, we initially estimated each observer's performance in a set of visual functions: the contrast sensitivity function (CSF), visual acuity (VA), the strength of the crowding (CW) and the influence of collinear and orthogonal flankers on the contrast detection of a central and vertically oriented Gabor patch of 4 cpd (Fig. 1). All stimuli were placed at 4° eccentricity, randomly either to the left or to the right with respect to a central fixation point. Subsequently, observers performed training sessions on the collinear configuration using different spatial frequencies across four target-to-flankers distances (from 2λ to 8λ), the same setup that we used in the pre-training sessions. We used a yes/no task and the psychophysical method of Constant Stimuli to estimate the contrast threshold values at which subjects perceived the target with a probability of 0.6 and 0.8. We aimed to compare the effect of the learning for these two contrast thresholds, since previous studies have shown that lateral interactions induce facilitatory modulations mainly at low contrast values [7], [8], [31]–[34].

## Results

The results of the present study suggest a different connectivity in the periphery of the visual field with respect to the fovea as well as a different training-dependent modulation of this connectivity that results in reduced suppression. Most importantly, we found that training improves contrast sensitivity and reduces crowding, whereas we did not find that learning transfers noticeable benefits to visual acuity.

### Lateral masking curves

**Figure 2:**
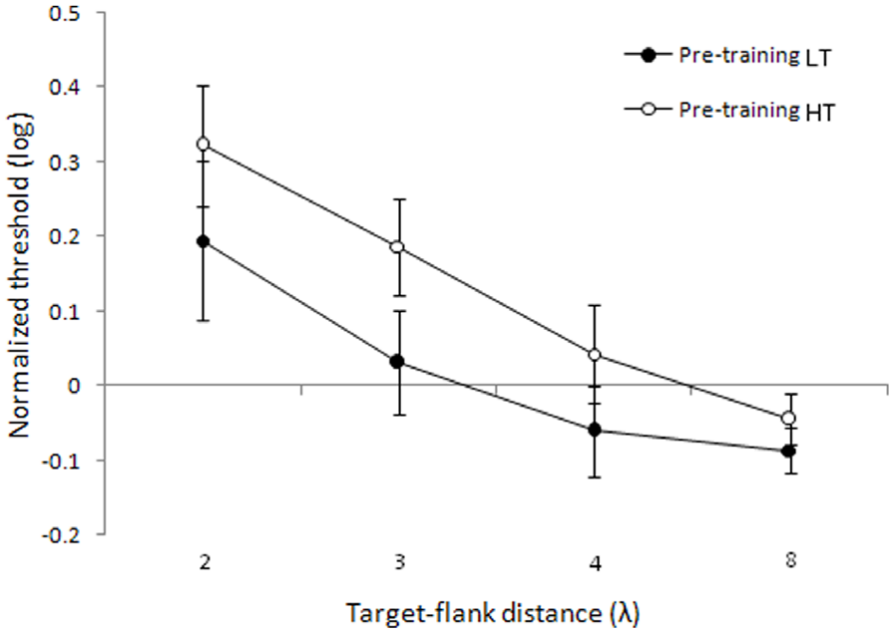
Detection thresholds for 0.6 and 0.8 probability levels. Thresholds corresponding to 0.6 probabilities (filled circles) and 0.8 probabilities (open circles), normalized by orthogonal flankers at 8λ as a function of the target-to-flanker distances (λ). Error bars ±1 s.e.m.

Fig. 2 shows the lateral masking curves that we derived from the pre-test contrast thresholds associated to either 0.6 (low contrast threshold - LT) or 0.8 (high contrast threshold - HT) detection probability versus target-to-flanker distances. Each contrast threshold was normalized by the baseline threshold for the orthogonally flanked target at a separation of 8λ. Lateral masking curves differ from those in the fovea in several aspects [5], [21], [24]; that is, at 4λ (a distance that produces consistent facilitation in the fovea), we did not find facilitation, which is in agreement with other studies [23]. Moreover, it should be noted that target-to-flankers separations of 3λ lead to inhibition instead of facilitation, as previously found [24]. The new result is that normalized LT reveal a collinear facilitation at a target-flanker distance of 8λ (*t*_7_ = −2.91, p = 0.023). The lateral masking curve referring to normalized HTs had a similar trend as the curve associated with LTs; however, in this case, we did not find any facilitation at 8λ (*t*_7_ = −1.33, p = 0.22).

Target suppression was found at a target-to-flanker distance that produces facilitation in the fovea (≈3λ), and this is compatible with the physiological finding that surround suppression increases with eccentricity [30]. Instead, the result that in the periphery LT reflects collinear facilitation at separations of 8λ was unexpected. This result suggests the presence of facilitatory lateral connections with larger extent in the near periphery respect to the fovea. The interpretation of this effect is not straightforward, because cell recordings showed that, in macaque area V1 at 2°–8° eccentricity, horizontal connections in layers II/III extend only 6±0.7 mm on average [35], whereas a human's V1 columns are only about twice the size of a macaque's V1 columns [36]. One possibility is that facilitation at such large separations is mediated by a cascade of long-range interactions [21]. Moreover, we only found facilitation at 8λ for the low contrast threshold, not for the high contrast threshold. This is consistent with the physiological finding that neuronal facilitation preferentially occurs when the collinearly flanked target is near its detection threshold [7], [8], [23], [31]–[33].

### Perceptual learning

**Figure 3:**
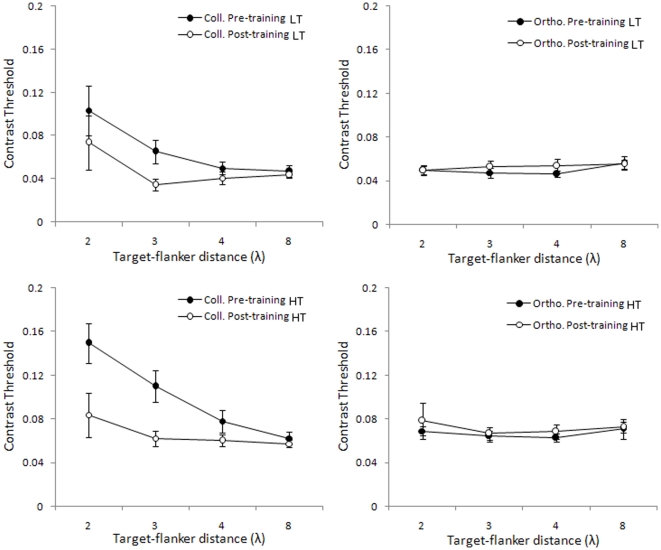
Contrast thresholds for target flanked by collinear and orthogonal flankers, before (pre) and after (post) training. Mean detection thresholds corresponding to 0.6 (top row) and 0.8 probabilities (bottom row), as a function of the target-to-flanker distances (λ), for the target flanked by collinear flankers (left column) or orthogonal flankers (right column). Data refer to Gabors with a spatial frequency of 4 cpd. Filled circles refer to pre-training measurements, and open circles refer to post-training measurements. Error bars ±1 s.e.m.

Training the contrast detection of a collinearly flanked target resulted in a significant decrease of contrast thresholds, but the learning effect did not transfer to the target of the same orientation and orthogonally oriented flankers (Fig. 3) Threshold reduction after training becomes more consistent as the target-to-flankers separation decreases in the range of 4λ - 2λ. Especially in the case of LT, the threshold significantly decreased at 3λ (*t*_7_ = 3.30, p = 0.013), whereas for the other target-to-flankers distances, we did not obtain any significant difference between the contrast thresholds measured in the pre and post-training sessions. In the case of HT, thresholds significantly decreased at 2λ (*t*_7_ = 3.38, p = 0.012) and at 3λ (*t*_7_ = 3.48, p = 0.010). At 8λ, where collinear flankers facilitate the observer's detection of the low-contrast target, training had no effect on either LT or HT. These results support the evidence that learning only reduces the suppression of the flankers [18].

**Figure 4:**
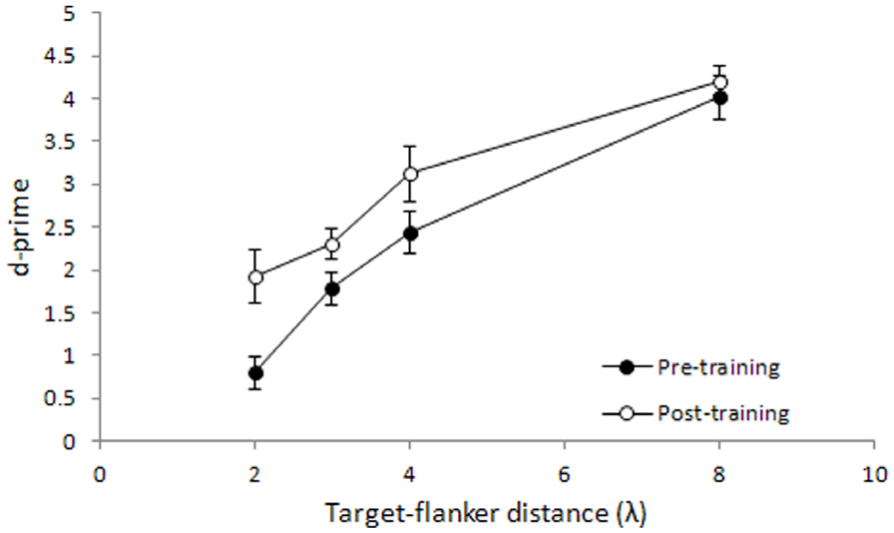
Detection sensitivity for target flanked by collinear flankers, before (pre) and after (post) training. Mean d-primes as a function of the target-to-flanker distances (λ) for the target flanked by collinear flankers. Data refer to Gabors with a spatial frequency of 4 cpd. Filled circles refer to pre-training measurements, and open circles refer to post-training measurements. Error bars ±1 s.e.m.

Since the yes/no procedure that we have used is sensitive to response bias [37], the procedure may have had a significant impact on the reported thresholds. To check for this possibility, we have reanalyzed the data by calculating *d*', which is a measure of sensitivity that is independent of bias. We calculated *d's* according to the Signal Detection Theory by using the accuracy data obtained in the catch trials (0.0 Michelson contrast) and in the highest contrast condition (0.1 Michelson contrast) at all target-to-flankers separations. The results appear in Fig. 4. The *d*' results reflect results obtained by measuring thresholds: sensitivity decreases progressively as λ decreases, and the effect of learning is only significant at 2λ (*t*_7_ = −2.64, p = 0.034) and nearly significant at 3λ (*t*_7_ = −2.11, p = 0.073); at 4λ and 8λ, where sensitivity is very high, there is no significant learning effect (*t*_7_ = −1.57, p = 0.16 and *t*_7_ = −0.75, p = 0.48, respectively).

Furthermore, the proportion of false alarms did not depend on whether the observer received training on any target-to-flanker distance (2λ: *t*_7_ = 0.02, p = 0.98; 3λ: *t*_7_ = 0.16, p = 0.87; 4λ: *t*_7_ = 0.068, p = 0.95; 8λ: *t*_7_ = 1.02, p = 0.34).

Perceptual learning improves visual performance in human adults, specifically for the trained task, pointing to plasticity in the adult visual cortex during training [36]. Several studies indicate a plasticity of lateral interactions that results from repetitive practice on detecting a flanked-target. The increased range of facilitatory interactions between target and flankers most likely reflects the effect of training [18] that produces a reduction in strength of short-range suppressive interactions between target and flankers [9]. We showed that perceptual learning with stimuli presented in the near periphery reduced short-range inhibition at 2λ and 3λ, but it did not increase facilitation. This is a new result: in fact previous studies [24] conducted with similar eccentricity, separations and spatial frequencies as those used in the present study did not find a consistent effect of training. This discrepancy may be due to the fact that we employed an appropriate number of sessions [25].

### Transfer of learning to orthogonally flanked Gabors

The high stimulus specificity observed in the learning studies [18], [38] points to an activity-dependent plasticity of the visual cortex, in which the specific interactions activated during training are modified to improve performance. We also confirmed the specificity of lateral interactions modulation, because we found an absence of a learning effect for the orthogonally flanked target that has the same orientation as the trained target. The lack of transfer of learning to a stimulus with the target having the same orientation but with flankers of different orientation suggests that perceptual learning affects not only the response of the individual underlying filter [39] but also its contextual modulation by co-axial filters outside its receptive field [6].

### Transfer of learning to a different global orientation of the collinear target-flankers configuration and to a different retinal position

**Figure 5:**
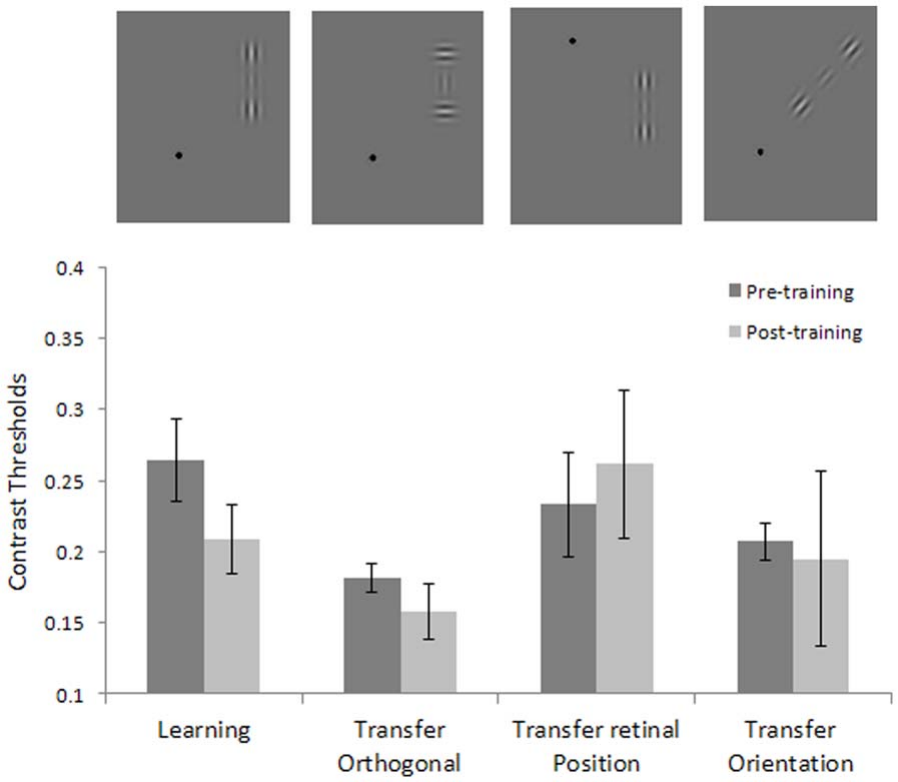
Specificity of learning: Contrast thresholds. Contrast thresholds measured before and after one week of training, with the target presented with collinear flankers (first and second column); contrast thresholds for untrained conditions with the target presented with orthogonal flankers (third and forth columns), with stimuli presented with different global orientations (fifth and sixth), and in different retinal positions (seventh and eighth) with respect to the trained condition. The data refer to a Gabor with a spatial frequency of 4cpd and a target-to-flankers distance of 3λ. At the top of the figure are illustrated examples of the stimuli used.

**Figure 6:**
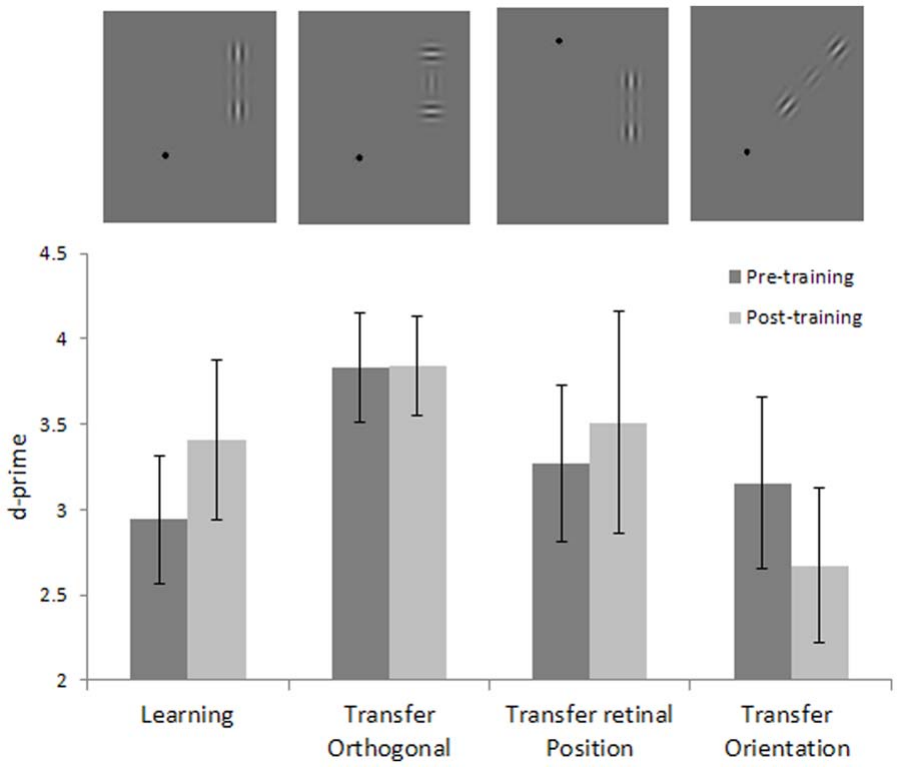
Specificity of learning: detection sensitivity. d-primes measured before and after one week of training with the target presented with collinear flankers (first and second column); contrast thresholds for untrained conditions with the target presented with orthogonal flankers (third and forth columns), with stimuli presented with different global orientations (fifth and sixth), and in different retinal positions (seventh and eighth) with respect to the trained condition. The data refer to the Gabor with a spatial frequency of 4cpd and a target-to-flankers distance of 3λ. At the top of the figure are illustrated examples of the stimuli used.

Since learning specificity is viewed as the main indicator of the level of processing at which learning takes place, we also tested the specificity of learning for target-flankers global orientation and for retinal position. We trained four new subjects for one week (1920 trials) in contrast detection of a collinearly flanked vertical target of 4 cpd with a target-flanker separation of 3λ. We found a significant learning effect (*t_3_* = 3.44, p = 0.04) obtained with the stimulus configuration, as presented randomly either in the upper-left or lower-right quadrant, but we did not find any transfer of learning to either the same stimulus presented in a symmetrical retinal location (either upper-right or lower-left, randomly) (*t*_3_ = −0.40, p = 0.71), nor to a 45 deg oriented collinear target-flankers configuration, presented in the same retinal position as the learning stimulus (*t*_3_ = −0.18, p = 0.87) (see Figs. 5 and 6).

The finding that these transfer stimuli are immune to perceptual learning of vertical orientations strongly suggests that the modulation of lateral interactions through perceptual learning is functionally specific. Ts'o and colleagues [40] have investigated the relationship between horizontal connections and the functional architecture of V1; their recording of the cell's activity demonstrated that the axon makes connections only with cells that have the same functional specificity (i.e., responsiveness to an iso-oriented line). Our results are compatible with their findings.

### Transfer of learning to CSF

**Figure 7:**
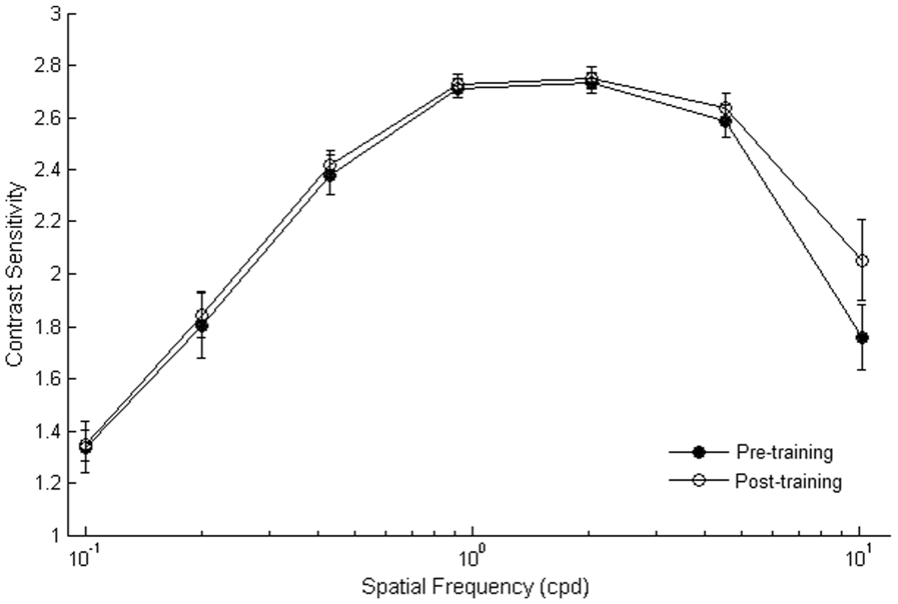
Contrast Sensitivity Functions, pre and post-training. Mean CSFs measured before training (filled circles) and after training (open circles). Sensitivity improved by a factor of ≈0.5 at the highest spatial frequency (10.2 cpd). CSFs were tested by using sine-wave gratings that varied in contrast and excluded the fovea (±4°). Error bars ±1 s.e.m.

Contrast sensitivity in the near-periphery was measured with standard methods before and after training in order to derive CSF for sinusoidal gratings at a range of spatial frequencies. Training lateral interactions increased contrast sensitivity only at the highest spatial frequency used (10.2 cpd) (*t*_7_ = −4.21, p = 0.004) (Fig. 7). It should be noted that the sensitivity for this spatial frequency is normally very low, at 4 deg of eccentricity.

Since CSF was tested at the same orientation as the collinearly flanked target Gabors, we do not know whether learning transfers at different orientations.

### Transfer of learning to VA

**Figure 8:**
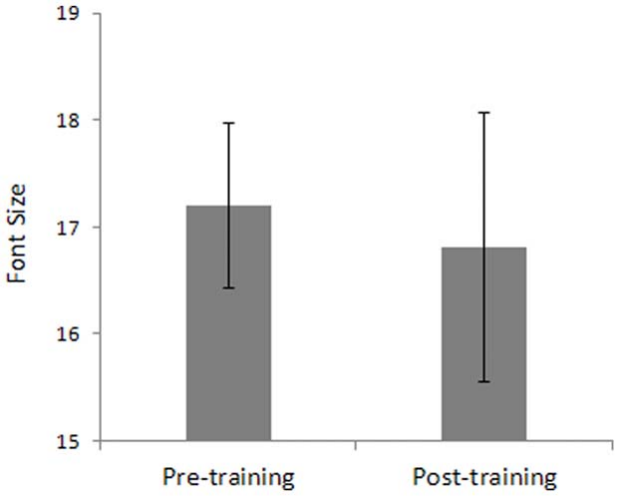
VA before training vs. VA after training. Mean VA is expressed as the font size that allowed 79% correct identification of a letter presented at 4° eccentricity. Error bars ±1 s.e.m.

Any incoming visual information is sampled by spatial filters in the visual cortex, and each filter is selective for a narrow range of spatial frequencies, the weak response of filters tuned for high spatial frequencies in the periphery is expected to limit VA. Thus, an improvement of the sensitivity for high spatial frequencies after the training period should improve peripheral VA. However, results (Fig. 8) showed that the reduction of suppressive lateral interactions after training did not improve VA in the peripheral visual field (*t*_7_ = 0.41, p = 0.69). A possible explanation to this result is that we trained only one orientation and we measured VA using alphabetic letters as stimuli that are made up of line segments with different orientations. It is possible that training had not generalized to every spatial orientation and therefore was not able to improve the observers' VA. Another possibility is that learning did not transfer to letter acuity because letter acuity depends on accurate encoding of a relative phase in addition to sensitivity to contrast, spatial frequency and orientation.

### Transfer of learning to CW

**Figure 9:**
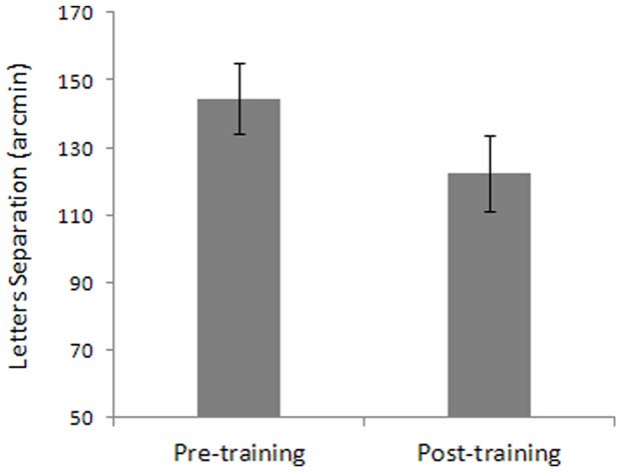
Results for the crowding (CW) test. CW is expressed as the distance (arcmin) between the target and the flankers letters. The target and flankers' font size corresponded to the font size threshold estimated in the VA task and increased by 20%. Error bars ±1 s.e.m.

Although training-dependent reduction of lateral suppression caused by collinear flankers at 2λ and 3λ had no effect on VA, it significantly reduced crowding in peripheral vision (*t*_7_ = 3.59, p = 0.009) so that the observer could better identify a target in a cluttered background (Fig. 9).

The dissociated effect of training on VA and CW may be a consequence of the fact that the strong lateral masking in the periphery is more likely to degrade identification when the target letter is surrounded by other letters rather than when the target letter is presented in isolation [27]. This masking phenomenon, known as crowding, increases with the eccentricity of the target, but it is relatively independent of the target's size [27]. Although many studies have claimed that CW reflects the combination of inappropriate features, the similar properties of surround suppression and crowding suggest that surround suppression may, at least in part, explain CW. Based on this assumption, it is not unlikely that the reduction of inhibitory lateral interactions has more effect on an observer's ability to identify crowded letters than on the observer's ability to identify single letters. Crowding is a peripheral phenomenon, so we do not expect it to be present in the fovea. However, letters to be identified are surrounded by other letters in the standard VA tests (ETDRS), so there is also the possibility that in previous studies, the effects of CW have been confounded with those of VA (for a review, see [27]). We did control for this confounding by measuring VA with only the letter-size as the dependent variable. Thus, we were able to dissociate the training's effect on VA from its effect on CW.

## Discussion

The results of this study suggest the presence of different lateral-interactions in the periphery with respect to the fovea. Suppressive interactions occurred at a larger range of target-to-flankers distances than in the fovea. Facilitation was found at larger separations than those at which the flankers affected the observers' detection of foveal targets. Moreover, we found that training lateral interactions at a range of target-to-flankers separations reduced suppression but did not increase facilitation. Most importantly, we found that learning reduced CW in addition to improving contrast sensitivity for high spatial frequencies, whereas it had no effect on VA.

The result that facilitation of target detection by the flankers occurs at large separations in the periphery seems to be incompatible with the finding of Angelucci and Bullier [35], who demonstrated that long-range connections in layers II/III of the macaques' striate cortex at 2°–8° eccentricity extend about 6±0.7 mm, whereas human striate cortex columns are about twice the size of the macaques' V1 columns [29]. Thus, it is possible that the larger extent of facilitatory lateral connections in the near periphery could be mediated by a concatenation of long-range interactions, as suggested by Polat and Sagi [21].

We also found that inhibitory long-range interactions were reduced by the training. A previous study [24] that investigated the effect of training lateral interactions in the periphery (4 deg), did not find consistent results (training reduced inhibition in only one subject). This inconsistency probably arose from the study's insufficient number of training sessions [25]. In contrast, by training subjects for 8 weeks (about 50 hours), the present study found a significant effect on the trained collinear flankers condition but no effect on the untrained orthogonal flankers condition. This last result is consistent with the selective effect of training on reducing suppressive lateral interactions, for it does not simply reduce contrast detection thresholds.

Previously, perceptual learning has been shown to be specific for the low-level trained stimulus and for the task [38]–[39], [41], suggesting modifications of neural processes at the primary visual cortex in adults. Perceptual learning has also been shown to be specific for collinear flankers. However, our results showed, in agreement with other findings [18]–[20], that systematic training in this low-level task yielded significant perceptual benefits to unrelated visual functions (e.g., crowding). How can the reduction of the strength of inhibitory low-level lateral-interactions explain the reduced crowding? Pelli et al. [42] argued that crowding reflects an excessive features integration process, so it is possible that the reduction in strength of the inhibitory long-range lateral-interactions at low-level may determine a more appropriate balance between inhibition and integration processes. Crowding for letters is likely to occur at the level of area V4, since it has been shown that macaques' receptive fields in V4 have an extension of about 0.5 ф (where ф represents the target eccentricity), which fits well with the extent of peripheral crowding for letters [43]. The effect of the training that we found on letter crowding may reflect the weakening of inhibitory long-range connections present at the level of area V1. However, it is not clear how reduced inhibition at low-level can modulate integration processes at higher levels. Cell recordings pointed out the existence of direct projections from V1 to V4 bypassing V2 [44]. It could be possible that the weakening of inhibitory long-range interactions after training at low-level might be forwarded to area V4 by exploiting secondary routes from V1. Although the inhibition of contrast detection by flankers and crowding are two distinct phenomena [26], [30], they may share the same first stage in which linear filtering processes take place. However, this hypothesis is disputable, because crowding occurs with suprathreshold stimuli [27]. Alternatively, it is possible that the lateral masking stimulus induces, instead of or in addition to feature learning, either location-learning [45] or rule-based learning [46] in a central site, which increases an efficient modulation of low and high-level inhibitory processes. More specifically, it is possible that learning occurs in a central site and consists of a reduction of inhibitory effects through external noise exclusion [47], both at a low and high level of processing. Indeed, the fact that spatial frequency, and target-flankers separation all varied during practice may have produced conditions that maximized the amount of transfer to new tasks. However, the lack of transfer to different retinal positions challenged this interpretation.

In conclusion, we showed that probing cortical interactions with a wide range of spatial frequencies and target-to-flankers separations could possibly modulate the spatial interactions in the peripheral visual field of normal sighted human adults. Most importantly, reduced lateral masking through perceptual learning in the periphery reduces crowding and consequently increases acuity for the target stimulus. Crowding is ubiquitous in spatial vision and occurs in a variety of tasks, including letter identification [48]–[50], vernier acuity [51]–[52], stereoacuity [53], and orientation discrimination [54]. By reducing crowding, perceptual learning may allow the periphery to perform several tasks in viewing conditions that are more similar to those present in central vision. This has important implications for the rehabilitation of low-vision patients who have lost the use of the fovea through macular degeneration, because these patients must exploit peripheral vision to perform tasks that normal sighted subjects perform in the fovea.

## Methods

### Apparatus

Stimuli were displayed on a 19-inch CTX CRT Trinitron monitor with a refresh rate of 75 Hz. The flankers and target stimuli were generated with the Matlab Psychtoolbox [55]–[56], whereas stimuli for VA and CW were generated using E-Prime software. The screen resolution was 1280×1024 pixels. Each pixel subtended ∼1.9 arcmin. We measured CSF by using sinusoidal gratings generated by a VSG2/3 graphics card. Gratings were displayed on a 17-inch Philips Brilliance 107P CRT monitor with a refresh rate of 70 Hz and a spatial resolution of 1024×768 pixels. We used a gamma-corrected lookup table (LUT) so that luminance was a linear function of the digital representation of the image.

### Subjects

Four authors and four naive subjects who were unaware of the purpose of the study participated in the experiments. Subjects sat in a dark room 57 cm from the screen. Viewing was binocular. They were instructed to fixate on a central fixation spot. All subjects had normal or corrected-to-normal visual acuity. All subjects gave their informed consent prior to their inclusion in the study. We have performed the study in accordance with the ethical standards laid down by the 1964 Declaration of Helsinki.

The study was approved by the Ethics Committee of the Department of General Psychology, University of Padua. We obtained written, informed consent from all participants involved in the study.

### Flanker and target stimuli

Stimuli were Gabor patches consisting of a cosinusoidal carrier enveloped by a stationary Gaussian. The mean luminance of the display was 46.7 cd/m_2_. Each Gabor patch was characterized by its sinusoidal wavelength λ, phase *ϕ*, and SD of the luminance Gaussian envelope (σ) in the (x, y) space of the image:
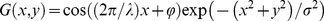
(1)In all experiments, σ = λ and *ϕ* = 0 (even symmetric). Gabors had a spatial frequency of 1, 2, 4, and 8 cpd. The location of the target relative to the fixation point (0.18 deg) was 4 deg either to the left or to the right. A vertical Gabor target (Figs. 1A and 1B, respectively) was presented flanked, above and below, by two high-contrast Gabor patches (0.6 Michelson contrast). During the learning session, the flankers were always vertically oriented and located at various distances from the target (i.e., 2λ, 3λ, 4λ, and 8λ). For spatial frequencies of 1, 2, and 4 cpd, we used target's contrast levels ranging from 0.016 to 0.1 (Michelson contrast) in steps of 0.2 log units, whereas for the spatial frequency at 8 cpd, the contrasts ranged from 0.023 to 0.59 (Michelson contrast) in steps of 0.35 log units. We used a different range of contrast levels for the higher spatial frequency to facilitate the detection of the stimuli. Moreover, we used an additional contrast level of 0.0 (Michelson contrast) in order to introduce “catch trials” to estimate the false alarms rate.

### VA and CW stimuli

The stimuli were 10 randomly chosen alphabet letters (D, N, S, C, K, R, Z, H, O, V) that were each presented for a duration of 100 ms. In the VA test, the location of the target letter was 4° either to the left or the right (randomly chosen trial by trial) with respect to the fixation point. The size of the letters varied according to a 1up/3down staircase [57]. The step size was 1 font size, the character type was Arial, and the starting font size was 20. Subjects had to say the letter displayed and the experimenter registered the answer. The session terminated after either 100 trials or 8 reversals. A threshold acuity, expressed as the font size for 79% correct identifications, was the mean of the 8 reversals.

In the CW test, the target letter was flanked on the left and the right sides by two different letters. The triplets could appear randomly either to the left or to the right of the fixation point, but the target letter was always at 4 deg from the fixation spot. In the CW test, the size of both the target letter and flanking letters was set 20% bigger than the VA threshold. Inter-letter distance varied according to a 1up/3down staircase [57]. The initial distance between letters was set at 95 arcmin, and the step size was constant at 1.9 arcmin. The session terminated either after 100 trials or 8 reversals. At the end of the procedure, we calculated the threshold by averaging the distance values in correspondence with the 8 reversals.

### CSF stimuli

We measured peripheral CSF with vertical gratings displayed on the whole screen area except for the fovea. This was carried out by placing a circular black spot (4° radius, the same eccentricity used for stimuli presentation in the training sessions) at the centre of the screen to force subjects to attend the near-periphery of their visual field while fixating on the center of the dark spot. Individual contrast thresholds were estimated with the Method of Limits over seven spatial frequencies (0.1, 0.2, 0.4, 0.9, 2.0, 4.5, and 10.2 cpd).

Fig. 5 shows the peripheral CSF obtained before learning (pre-training) and after learning (post-training).

### Procedure

Contrast sensitivity functions (CSF), visual acuity test (VA) and crowding test (CW), in addition to contrast thresholds for the lateral interaction task, with both collinear and orthogonal flankers, were measured initially to establish individual baseline performances and after the training period. We tested lateral interactions by comparing the contrast detection of a vertical Gabor target (4 cpd) flanked by either two vertically oriented Gabor patches (collinear condition – Fig. 1A) or two horizontal Gabors (orthogonal condition – Fig. 1B) with target-to-flankers distances of 2λ, 3λ, 4λ, and 8λ. The contrast detection threshold was measured for the target Gabor presented at 4 deg of eccentricity. Each stimulus was presented for 133 ms.

A standard training block consisted of a contrast-detection task on the central Gabor patch flanked by two high-contrast and collinear Gabor patches. A typical daily session consisted of four blocks, in which the target-to-flankers distance varied, starting from the highest distance (8λ). A weekly session consisted of four consecutive daily sessions. The spatial frequency of the Gabor patches varied between daily sessions, starting from the lowest spatial frequency (1 cpd). Each experimental block consisted of 96 randomly presented trials that corresponded to 8 repetitions of 12 stimulus conditions: 6 (contrast levels)×2 (spatial positions). The Method of Constant Stimuli and a yes/no detection paradigm were used. Thus, a standard daily session comprised 384 trials separated in four blocks, each dedicated to one target-to-flankers distance. Globally, each subject performed 160 sessions distributed over the course of 8 weeks. A logistic function was fitted to the data in order to estimate the contrast thresholds at which the subjects detected the target with a probability of 0.6 and 0.8.
